# Evaluating Pillar Industry’s Transformation Capability: A Case Study of Two Chinese Steel-Based Cities

**DOI:** 10.1371/journal.pone.0139576

**Published:** 2015-09-30

**Authors:** Zhidong Li, Dora Marinova, Xiumei Guo, Yuan Gao

**Affiliations:** 1 School of Management, Hefei University of Technology and Key Laboratory of Process Optimization and Intelligent Decision-making, Ministry of Education, Hefei, China; 2 Curtin University Sustainability Policy (CUSP) Institute, Curtin University, Perth, Australia; Southwest University, CHINA

## Abstract

Many steel-based cities in China were established between the 1950s and 1960s. After more than half a century of development and boom, these cities are starting to decline and industrial transformation is urgently needed. This paper focuses on evaluating the transformation capability of resource-based cities building an evaluation model. Using Text Mining and the Document Explorer technique as a way of extracting text features, the 200 most frequently used words are derived from 100 publications related to steel- and other resource-based cities. The Expert Evaluation Method (EEM) and Analytic Hierarchy Process (AHP) techniques are then applied to select 53 indicators, determine their weights and establish an index system for evaluating the transformation capability of the pillar industry of China’s steel-based cities. Using real data and expert reviews, the improved Fuzzy Relation Matrix (FRM) method is applied to two case studies in China, namely Panzhihua and Daye, and the evaluation model is developed using Fuzzy Comprehensive Evaluation (FCE). The cities’ abilities to carry out industrial transformation are evaluated with concerns expressed for the case of Daye. The findings have policy implications for the potential and required industrial transformation in the two selected cities and other resource-based towns.

## Introduction

China’s massive economic growth increased demand for natural resources beyond human imagination [1] and resource-based cities played an important part in delivering this. National large-scale steel bases in China are located in 27 out of the country’s 34 administrative divisions at provincial level. These steel bases are dispersed among 30 cities, more than half of which are considered steel-based and dependent on iron and other natural resources. According to China’s official government document *Sustainable Development Plan of Resource-based Cities (2013–2020)* [2], the most steel-dependent cities are Anshan, Benxi, Panzhihua, Baotou, Maanshan, Daye, Hnadan, Laiwu, Lingyuan and Liuzhou. These cities serve as steel-suppliers for the whole of China and are prominent contributors to the development of the Chinese steel and other related industries. Nevertheless, many of these settlements have gradually descended to being over-dependent on this resource exhibiting a simple economic structure due to their geographic location, urban economy, national policies and development planning. Consequently, they are trapped in a vulnerable steel industry, imbalanced economic structure, inactive alternative industries, unemployment [3], poverty and serious environmental destruction in the process of urban development [4]. More than 90 per cent of these cities are confronted with the need for urban and economic transformation [5].

Within China’s regionally imbalanced economy [6], resource-based cities have been on a gradually degrading trajectory from petroleum to mixed resources, from metal to coal [7]. Since 2013 they are firmly on China’s government policy agenda for securing national energy and resources, transforming the economic development pattern, promoting the new ways of industrialization and urbanization and building a resource-conserving and environment-friendly society [2]. These cities are expected to become resilient and sustainable themselves [2,8]. What is the ability of China’s steel-based cities to undertake a most needed transformation and continue to provide employment and other opportunities to their residents? Globally there are examples of successful industrial transformation of steel-based cities, such as Pittsburgh in USA, Ruhr in Germany and Lorraine in France [9] but would the Chinese cities have the capacity to shift towards a more sustainable trajectory? There is an urgent need to understand the differences in development and capabilities of resource-based cities in China.

This study analyses the potential for industrial transformation using two case study examples from China. The following section examines existing theories and literature in relation to urban life cycles, urban transformation and previous research on Chinese resource-based cities. A conceptual model for analyzing industrial transformation is then presented. The model is further developed by establishing a system of indicators for evaluating the capability for transformation of the resource city’s pillar industry, the city itself and any industries that could substitute the pillar industry. Fuzzy Comprehensive Evaluation (FCE), text mining and Delphi expert evaluation are explained and used to create the evaluation system. The industrial restructuring of the steel-based Chinese cities of Panzhihua and Daye is examined providing a comparison between the two cases and policy recommendations.

## Theories and Literature Review

Two theories are of particular interest to the way cities evolve, namely life cycle and urban transformation theory. They are discussed below and an overview of previous research in relation to Chinese cities is presented.

### Life cycle theory

Resource-based towns or cities (we prefer cities when referring to Chinese settlements because of their size) are those developed around the discovery, extraction, transportation and processing of mineral and oil deposits, agricultural commodities, timber and forest products, fishing or hydro-electrical power. These settlements usually depend on a single industry [10]. Many of them are associated with mining, petroleum and other related industries including heavy industries, such as metallurgy, refining and steel production. The resource-based cities emerged in response to the strong demands for resources of the industrializing and globalizing national and world economies [11,12]. They are common for developed countries such as in Europe [13], USA, Australia and Canada, but also in emerging and developing economies, such as China, South Africa and in Latin America. These towns share many common characteristics, linked to the fact that they are controlled by one industry the demands for whose products are determined somewhere else [14]. Since the mid-1990s with the advent of the knowledge economy, there has been a lot of pressure on these settlements to diversify and transform their economies to be better positioned for the future [15].

Back in the 1970s, four stages were identified in the development of single industry based towns, including resource-based cities, namely: construction, recruitment, transition and maturity [10]. Bradbury and St. Martin extended the four-stage theory by adding two more phases, related to the end of life of these cities, namely: decline and closure [16]. Faced with the depletion of the original mineral resources, commodity price fluctuations (particularly during recession), competition from other resource suppliers and geographic locations, the town or city has the prospect to either witness the consequences from the dying of its single pillar industry and out-flow of population or take actions towards transformation.

### Urban transformation theory

Since the 1980s, technology and innovation proved to be the main driving forces of economic growth. Even resource-based economies, such as Australia and Canada, have become technology-intensive and capital-intensive. Describing the transition process of Canadian resource-based cities, faced with depletion of mining and petroleum resources in the mid-1990s, Barnes et al. argue that the application of new technologies leads to capitalization and scale development in the resource industries [17]. They undergo industrial transformation by becoming more technologically sophisticated. Some Canadian resource-based towns were able to take advantage of this process and build new pillar industries while others have fallen into decline [17]. In the case of Canada again, Parker found that cities do not reduce their dependency on resources or increase their high value-added industrial activities even when there is higher stability in the exports of resource-based products and reduced volatility of commodity prices [18]. Urban transformation seems to happen through various strategies of entrepreneurialism, including development of tourism, creative industries, retirement and pension industries establishing a post-productive city model. However, many cites fail to achieve such a transformation [19].

Several studies exist which develop sustainability indicators for resource-based cities, such as direct and indirect energy use, resource consumption, dependence and interference [20], socio-economic indicators, residents’ living standard and effectiveness of government policies [21], distinction between the performance and collaboration of internal and external systems [22]. A comprehensive evaluation framework for assessing the resource-based cities’ capacity to transform themselves however is yet to be established which is a major goal of this study.

### Previous research on resource-based Chinese cities

China’s resource-based cities have also been subject of investigation from different perspectives briefly summarized below:

- *Domino Effect* [23]—this approach regards the declining of the pillar industry as a result from resource depletion as the fundamental reason for the recession of a resource-based city [24]. The locking effect of the single industry within the resource-based cities triggers a downturn in the resource-oriented enterprises [25], hence a domino effect.
- *Diversified Transformation—*the resource-oriented city becomes more integrated locally becoming the production base of raw materials and growth center for the regional economy [26]. Consequently, through diversification these settlements can transform into modern industrialized cities featuring better structure, complete functions, distinctive features and high competitiveness [27].
- *Impact Factors—*a series of factors, such as urban spatial distribution, production technologies, operating mechanisms, sales markets, social settings and other major contributors [28], influence the transition of the resource-based cities. When the interactions between these factors are restructured in innovative ways within the urban system, this allows the resource-based city to transform its economy [29].
- *Integrated Strategy Model—*the model emphasizes the parallel development of industrial extension, upgrade and diversification, which are based on integrating government leadership and market regulation [30]. The continued existence of the resource-based cities is achieved through diversified development, moderate resource exploitation and other strategies.
- *Sunk Cost and Profit Maximization*–the cost of transforming resource-based cities has been examined through two different perspectives: the corporate viewpoint aimed at profit maximization and the government perspective targeting steady transformation [31]. The difficulty of transformation is acknowledged as economic and social sunk costs, which can be compensated and reduced through the market mechanism and/or administrative power [32].
- *Industrial Ecology Transformation—*the transformation of resource-based cities is seen as adopting the principles of industrial ecology [33] which allow a transition to symbiosis between different industries and creating a circular economy with no or little waste [8,34,35] and potential for low-carbon industries [36].

Although some previous studies have explored economic transition policies [37], the actual ability of such settlements to transform has rarely been assessed in previous analyses. This capacity however is the key factor in selecting new substitute industry/ies and is also the basis for successful transformation. Furthermore, a review of studies on the development of China’s resource-based cities points out that qualitative and descriptive analysis prevails and calls for methods which combine quantitative and qualitative approaches [11]. Combining statistical data and expert evaluation, this paper develops a system of indicators for a quantitative assessment of the transformation ability of steel-based cities and then provides advice on whether to develop the new substitute industries. It integrates Fuzzy Comprehensive Evaluation (FCE) and Analytic Hierarchy Process (AHP) techniques. The original Fuzzy Relation Matrix (FRM) created during the application of FCE only with the Expert Evaluation Method (EEM) [38,39] is improved by including objective statistical data. The qualitative EEM is used to finalize the indicators which are then ranked according to the AHP. This heightens the impartiality of the assessment and reduces subjectivity.

## Analyzing Industrial Transformation

**Fig 1:**
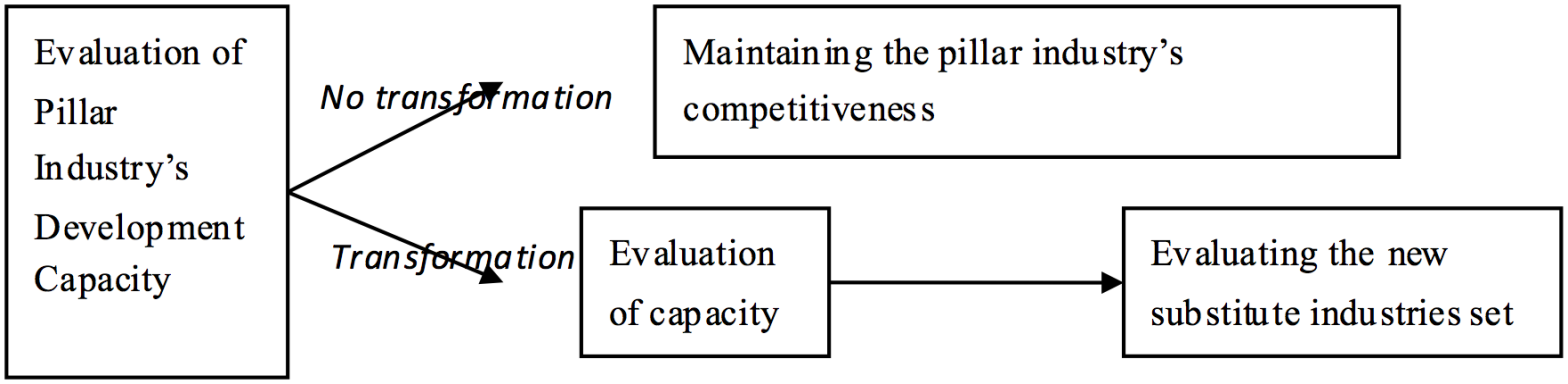
Evaluating the Transformative Capacity of the Pillar Industry.

The evaluation of the transformative capacity of the primary industry (we use “primary”, “pillar” and “single” as synonyms in relation to the main industry) in a resource-based city, a steel-based city in this case, includes three steps (see Fig 1). First, the need for industrial transformation is examined within the specific developmental circumstances of the steel-based city with the two options of maintaining and transforming the evaluated pillar industry. If the city can maintain a competitive steel-industry, it would not be necessary for it to convert to a different industry or diversify. If this is not the case, the capacity for industrial transformation needs to be evaluated. The third step is to identify new alternatives to substitute the steel industry.

## Evaluation System for Urban Industrial Transformation Capacity

As captured in the above literature review, publications on China’s resource-based cities have appeared in international journals in recent years. Nevertheless, their number remains small and the bulk of research, government information and news reports on this subject is still in Chinese periodicals, books, websites and other media. The various Chinese national databases contain rich material specific to topics, such as resources development, urban and regional planning, urban industrial capacity, environmental protection and transition to a more sustainable ways of production and consumption, which is related to the local context, research findings, practical experience and policies. In fact, most of the foreign English-language publications capture issues that are also discussed in domestic and national outlets. Furthermore, important English language articles are often translated into Chinese and covered by the national databases. Similarly to this study, many Chinese papers and reports contain international references. The rich and specific information contained in the national databases allowed us to opt for basing the research on Chinese sources while being aware of existing indicators and previous evaluation of resource-based cities in the international literature.

Based on the literature review, text-mining and expert evaluation, this study establishes an indicator system for evaluating the transformation of Chinese steel-based cities. The evaluation is applied to the existing pillar industry, the city’s transformation capability and the development capacity of substitute industries. Particular attention is paid on increasing the evaluation objectivity, and hence reducing the subjectivity of expert evaluation, and overall enhanced reliability of the evaluation outcome. The method for selecting the indicators as well as the actual indicator system and evaluation method are described below.

### Indicator selection

The following two methods for selecting the evaluation indicators are used, namely text mining and expert evaluation:

*• Text Mining Method—*this is the process of extracting text features through text segmentation techniques [40]. Specifically, this study derives the 200 most frequently used words from 100 available publications and papers related to steel- and other resource-based cities through keywords, verbal associations, similar and reverse retrievals using the Document Explorer technique [40].

The 100 available publications and papers were identified in the following way:

1. 57 papers were obtained through searching the China National Knowledge Infrastructure (CNKI) academic database website cnki.net (http://www.cnki.net/) with the following keywords: “resource-based city”, “development” and “industry”. The CNKI database is a key e-publishing project of China which includes e-journals, newspapers, dissertations, proceedings, yearbooks and other reference works.
2. 29 articles were obtained with the keywords “steel”, “city” and “development” from China’s news search engine (http://news.chinaso.com) which is the country’s most authoritative and widely used search platform, composed of seven official newspapers.
3. 14 more articles (two per city) were selected with the same news search engine (http://news.chinaso.com) by pairing the names of the seven resource-based cities (Anshan, Benxi, Panzhihua, Baotou, Maanshan, Daye and Liuzhou) and “economic development” as keywords.

The Text Mining process extracted text features through text segmentation techniques around nouns and verbs, using the IK Analyzer for Chinese words (https://code.google.com/p/ik-analyzer/) which eliminated pronouns, adverbs, quantifiers, adjectives, conjunctions and syndetic words. The IK Analyzer derived the 200 most frequently used words or short phrases from the 100 identified publications and papers. The text mining method allowed extracting the feature words to determine and describe the indicators according to the computer statistics about frequencies of appearance.

The process of extracting the high-frequency words through text mining provides information about expectations by the public, government and academia in relation to the development of resource-based cities. Establishing an indicator system through text mining in fact combines qualitative and quantitative approaches. To some extent it can also correct for differences in knowledge, subjective opinion and personal creativity among experts and scholars. The method of text mining hence enhances qualitative content analysis allowing for quantitative computing characteristics.

*• Expert Evaluation Method—*the popular Delphi method is used to identify the individual indicators and indicator system using the 200 words identified with IK Analyzer. This enhances the quantization process of text mining which produced feature words extracted by the computer.

The 200 words were given to selected experts who were asked to list their chosen keywords as two level indicators—more general at level 1 and more detailed at level 2 developing a typology and creating an indicator system for the transformation and development of resource-based cities.

Three experts—a data mining expert, an expert in researching resource-based cities and a risk investment analysis expert, were invited to review the 200 words in order to simplify the set and determine the key expressions. Their selection was based on previous experience, research standing and work-related credentials. The Delphi method allowed structured communication which required the experts to conduct the evaluation independently from and without making contact with each other until agreement was reached.

Once the keywords identified by the experts were received, the authors selected 53 indicators based on them and established an indicator system which reflects the characteristics of steel-based cities, applicability of FCE and data availability. The expert input helped also to eliminate similar indicators, add any missing indicators and make adjustments according to perceived importance.

### Indicator System

The indicator system includes three groups according to the steps of the evaluation procedure (refer to Fig 1). Group 1 (see Table 1) reflects the capacity of the existing single industry in the Chinese city to be maintained which means examining whether the pillar industry, in this case steel, needs to be converted or not [41]. The second group of evaluation indicators (see Table 2) refers to the capacity for industrial transformation with measures at two levels—more general and specific. Group 3 (see Table 3) covers indicators for selecting alternative industry/ies. According to China Industry Economy Statistical Yearbook [42], eleven potential new industries are considered: agro-industry, food manufacturing, pharmaceuticals, fabricated metal products and seven high-tech industries (namely general equipment manufacturing, equipment manufacturing, transportation equipment manufacturing, electrical equipment manufacturing, computers software and information services, instrumentation and equipment manufacturing for cultural products, logistics and other business services). These 11 industries are viewed as a new industrial set about which the experts decide whether there are viable alternative substitutes having in mind the specific conditions of the selected cities. Then FCE is then applied to evaluate the substitute industries [43].

**Table 1:** Evaluation Indicators for Existing Pillar Industry’s Capacity.

Object	Weight	Indicators	Evaluation Method Category
Existing Industry’s Transformative Capacity	*a*_1_	Ratio of Patents per Existing Pillar Industry Enterprise	*T4*
	*a*_2_	Mineral Reserves	*T3*
	*a*_3_	Rate of Profit in the Existing Pillar Industry	*T2*
	*a*_4_	Growth Rate of the Existing Pillar Industry’s Products	*T4*
	*a*_5_	Existing Pillar Industry Production	*T4*
	*a*_6_	Ratio of Investment in Industrial Pollution Control to Industrial Pollution Emission	*T4*

**Table 2:** Evaluation Indicators for the Steel-Industry’s Transformational Capacity.

Weight	First Level	Weight	Second Level	Evaluation Method Category
A_1_	Contribution to City Development (*U*_1_)	*a*_11_	Growth Rate of GDP	*T1*
		*a*_12_	Regional GDP per Capita	*T1*
		*a*_13_	Industrial Structure	*T3*
		*a*_14_	Household Savings per Capita	*T1*
		*a*_15_	Educational Expenditure per Capita	*T1*
		*a*_16_	Residential Consumption per Capita	*T1*
A_2_	Industrial Innovation Capability (*U*_2_)	*a*_21_	Share of High-tech Industries Output in GDP	*T1*
		*a*_22_	Ratio of Investment in Science and Technology to GDP	*T1*
		*a*_23_	Ratio of Patents to Number of Enterprises	*T1*
		*a*_24_	Ratio of Foreign Capital to Total Investments	*T1*
A_3_	Environmental Protection Ability (*U*_3_)	*a*_31_	Pollution Discharge (wastewater and waste emissions) per GDP —	*T1*
		*a*_32_	Ratio of Completed Investment in Industrial Pollution Control to Industrial Discharge	*T4*
		*a*_*33*_	Environmental Planning and Law Enforcement	*T3*
		*a*_34_	Energy Consumption per GDP —	*T2*
A_4_	Industry Synergy Ability (*U*_4_)	*a*_41_	Government Ability in Development Planning	*T3*
		*a*_42_	Synergistic Ability among Industries	*T3*
		*a*_42_	Allocating Efficiency of the City’s Industries	*T3*
A_5_	Strength of the Pillar Industry (*U*_5_)	*a*_51_	Fixed Investment in the Existing Industry —	*T2*
		*a*_52_	Employment in the Existing Industry —	*T2*
		*a*_53_	Products of the Existing Industry —	*T2*

Note: Indicators marked with “—”are negatively correlated.

**Table 3:** Evaluation Indicators for Substitute Industries’ Development Capacity.

Object	Weight	Indicators	Evaluation Method Category
Ability of New Industry to Replace Existing Pillar Industry	*a*_1_	Fixed Investment in Substitute Industries	*T2*
	*a*_2_	Government Investment and Fiscal Subsidy	*T3*
	*a*_3_	Employment in Substitute Industry	*T2*
	*a*_4_	Income per Capita of Substitute Industry Employees	*T4*
	*a*_5_	Output of Substitute Industry	*T2*
	*a*_6_	Profit Rate of Substitute Industry	*T4*
	*a*_7_	Degree of Similarity between the Existing and Substitute Industries	*T3*
	*a*_8_	Prospects of Substitute Industries	*T3*

### Evaluation Method

The developed evaluation system includes indicators at one—Tables 1 and 3, and two levels–Table 2. From the point of view of their values, some indicators are quantitative and others qualitative. A multilevel FCE is well-suited to be used for constructing the evaluation model [44]. Using Table 2 (which contains more complex indicators) as an example, the evaluation model is explained below. It includes the following steps:

### Determining the evaluation set

The evaluation set is determined as *V* = {*v*_1_, *v*_2_, … *v*_5_}, representing respectively “excellent”, “good”, “average”, “worse” and “bad”. Preference is given to the use of objective data and quantifiable indicators but this is not always possible. The first-level indicators are categorized into four categories—T1, T2, T3 and T4.

Category T1 refers to the value of a particular indicator in the resource-based city compared with other cities nationally. The value *v* (“excellent” to “bad”) is determined based on calculating a ratio as explained below.

*Q*_i_(*i* = 1, 2, … 32) is the value of the indicator for each province (28 provinces and 4 municipalities in total);

*Q*_0_ is the national value of the indicator;

*Q* is the selected resource-based city’s value of the indicator.

If $Z_{1}=\frac{Q_{0}-Min\left(Q_{i}\right)}{2.5}$ and $Z_{2}=\frac{Max\left(Q_{i}\right)-Q_{0}}{2.5}$, the [*Z*_1_, *Z*_2_] interval is subdivided into five sub-intervals by using the national value *Q*_0_ as the reference point. Table 4 shows the respective values for *v* depending on the value of Q for the particular resource-based city; Table 5 shows the respective values when there is a negative link between the second-level and the first-level indicators (as is the case with pollution discharge per GDP).

**Table 4:** Values for T1 Indicators (Positive Relationship).

*Q* ⊆ (−∞, Q_0_ − 1.5*Z*_1_)	corresponds to	*v*_5_
*Q* ⊆ (*Q*_0_ − 1.5*Z*_1_, *Q*_0_ − 0.5*Z*_1_)	corresponds to	*v*_4_
*Q* ⊆ [*Q*_0_ − 0.5*Z*_1_, *Q*_0_ + 0.5*Z*_2_]	corresponds to	*v*_3_
*Q* ⊆ (*Q*_0_ + 0.5*Z*_2_, *Q*_0_ + 1.5*Z*_2_]	corresponds to	*v*_2_
*Q* ⊆ (*Q*_0_ + 1.5*Z*_2_, *Q*_0_ +∞]	corresponds to	*v*_1_

Note: The value of Q is at the city level while Qi are at provincial level due to data availability. Consequently, it may be the case that Q < Min (Q_i_) or Q > Max (Q_i_). Therefore the minimum and maximum of the data are shown as −∞and +∞, which do not exist in reality.

**Table 5:** Values for T1 Indicators (Negative Relationship).

*Q* ⊆ (−∞, Q_0_ − 1.5*Z*_1_)	corresponds to	*v*_1_
*Q* ⊆ (*Q*_0_ − 1.5*Z*_1_, *Q*_0_ − 0.5*Z*_1_)	corresponds to	*v*_2_
*Q* ⊆ [*Q*_0_ − 0.5*Z*_1_, *Q*_0_ + 0.5*Z*_2_]	corresponds to	*v*_3_
*Q* ⊆ (*Q*_0_ + 0.5*Z*_2_, *Q*_0_ + 1.5*Z*_2_]	corresponds to	*v*_4_
*Q* ⊆ (*Q*_0_ + 1.5*Z*_2_, *Q*_0_ +∞]	corresponds to	*v*_5_

Category T2 is the Location Quotient (LQ) which reflects the concentration ratio of the given indicators within the resource-based cities [45]. It is calculated using the formula $L_{mn}=\frac{e_{mn}/e_{n}}{E_{mn}/E_{n}}$ where *L*_mn_ is the LQ for industry m in city *n*;

*e*_n_ is the value of the given indicator in city *n*;

*e*_mn_ is the value of the given indicator for industry m in city *n*;

*E*_n_ is the national value of the given indicator;

*E*_mn_ is the national value of the given indicator for industry m;

$L_{mn}^{i}\left(i=1,2,\cdots 32\right)$ is the LQ of the given indicator for all industries in province i (28 provinces and 4 municipalities in total).

If $Y_{1}=\frac{1-Min\left(L_{mn}^{i}\right)}{2.5}$, $Y_{2}=\frac{Max\left(L_{mn}^{i}\right)-1}{2.5}$, the indicators correspond to different evaluation values as shown in Table 6.

**Table 6:** Values for T2 Indicators.

*L*_mn_ ⊆ (−∞, 1 − 1.5*Y*_1_)	corresponds to	*v*_5_
*L*_mn_ ⊆ (1 − 1.5*Y*_1_, 1 − 0.5*Y*_1_)	corresponds to	*v*_*4*_
*L*_mn_ ⊆ [1 − 0.5*Y*_1_, 1 + 0.5*Y*_2_]	corresponds to	*v*_3_
*L*_mn_ ⊆ (1 + 0.5*Y*_2_, 1 + 1.5*Y*_2_]	corresponds to	*v*_2_
*L*_mn_ ⊆ [1 + 1.5*Y*_2_, 1 +∞]	corresponds to	*v*_1_

Category T3 consists of qualitative indicators. Their affiliation with the evaluation set is examined using EEM [38,39]. Values from *v*_1_ to *v*_5_ are allocated by surveying nine experts (the original three plus six new experts—one each from text mining and risk investment and four from economic development of resource-based cities) who gave the indicators a qualitative strength levels on the scale of “excellent”, “good”, “average”, “worse” and “worst”. The obtained evaluation is then used in the assessment process.

Category T4 is based on comparison between the values for a given industry and the other industries in a resource-based city, namely:

*F* is the indicator value for the given industry;

*F*_*i*_(*i* = 1,2…*n*) indicator value for the other industries.

If $X=\frac{Max\left(Q_{i}\right)-Min\left(F_{i}\right)}{5}$, the corresponding evaluation values are shown in Table 7.

**Table 7:** Values for T4 Indicators.

*F* ⊆ [*Min*(*Q*_*i*_), *Min*(*Q*_*i*_) + *X*)	corresponds to	*v*_5_
*F* ⊆ [*Min*(*Q*_*i*_) + *X*, *Min*(*Q*_*i*_) + 2*X*)	corresponds to	*v*_4_
*F* ⊆ [*Min*(*Q*_*i*_) +2*X*, *Min*(*Q*_*i*_) + 3*X*)	corresponds to	*v*_3_
*F* ⊆ [*Min*(*Q*_*i*_) + 3*X*, *Min*(*Q*_*i*_) + 4*X*)	corresponds to	*v*_2_
*F* ⊆ [*Min*(*Q*_*i*_) + 4*X*, *Max*(*Q*_*i*_))	corresponds to	*v*_1_

### Determining the weights of the indicators

Table 2 includes indicators at two levels: *U* and its sub-indicators *U*_*i*_. A judgment matrix *A*_*i*_ is established based on the indicator weights by employing AHP [46,47,48] and pairwise comparison [49].

$$A_{i}=\left[\begin{array}{cccc} a_{11} & a_{12} & \cdots & a_{1n}\\ a_{21} & a_{22} & \cdots & a_{2n}\\ \vdots & \vdots & \ddots & \vdots \\ a_{n1} & a_{n1} & \cdots & a_{nn} \end{array}\right],\;a_{ii}=1,a_{ij}=\frac{1}{a_{ji}}\left(i\neq j\right)$$

The Consistency Ratio (CR) is used to measure the consistency of the judgment matrix A_i_. It satisfies the consistency requirement when

$$CR=\frac{CI}{IR}=\frac{\left(\lambda_{max}-n\right)/n-1}{IR}\leq 0.1\;$$

(*λ*_*max*_ is the maximal eigenvalue of the matrix.).

The method adopted for the eigenvector is as follows. If $M_{i}=\overset{n}{\underset{i=1}{\prod }}a_{ij}\left(i=1,2,\cdots n\right)$, $\bar{W_{i}}=\sqrt[{n}]{M_{i}}$ *, $\bar{W_{i}}={\left[\begin{array}{cccc} \bar{W_{1}} & \bar{W_{2}} & \cdots & \bar{W_{n}} \end{array}\right]}^{T}$* is normalized as $W_{i}=\frac{\bar{W_{i}}}{\sum_{j=1}^{n}\bar{W_{j}}}$. Then *W* = [*W*_1_ *W*_2_ … *W*_*n*_]^T^ is the required eigenvector. If *A = W^T^* = [*W*_1_ *W*_2_ … *W*_*n*_], A is the weight of the indicators. Subsequently, the weight of the different indicators is calculated through pairwise comparisons conducted by experts and using AHP.

The weight vectors for the respective indicators are:

- Table 1: A′ = [*a*_1_ *a*_2_ *a*_3_ *a*_4_ *a*_5_ *a*_6_] = [0.0425 0.2516 0.3806 0.0643 0.1009 0.1602],
*λ*_*max*_ = 6.1223, *CI* = 0.0245, *IR* = 1.26, *CR* = 0.0194
- Table 2: A″ = [*a*_1_ *a*_2_ *a*_3_ *a*_4_ *a*_5_] = [0.2589 0.3800 0.1711 0.0770 0.1131]
*λ*_*max*_ = 5.1333, *CI* = 0.0333, *IR* = 1.12, *CR* = 0.0298

The weight of second level indicators in Table 2 are shown in Table 8.

- Table 3: A‴ = [*a*_1_ *a*_2_ *a*_3_ *a*_4_ *a*_5_ *a*_6_ *a*_7_ *a*_8_] = [0.1065 0.0714 0.0479 0.0327 0.2319 0.3280 0.0231 0.1585]
*λ*_*max*_ = 3.1782, *CI* = 0.0136, *IR* = 0.38, *CR* = 0.0357

where:

A′ represents the calculated capacity of the existing pillar industry;

A″ represents the calculated Steel-Industry’s Transformational capacity; and

A‴ represents the calculated capacity of Substitute Industries’ development.

**Table 8:** Weight of Second Level Indicators from Table 2.

*A*_*i*_	Weight	*λ*_*max*_	*CI*	*IR*	*CR*
*A*_1_	[0.3415 0.2824 0.1585 0.0979 0.0667 0.0529]	4.2199	0.0251	1.26	0.0199
*A*_2_	[0.4820 0.1170 0.1831 0.2178]	6.1254	0.7332	0.89	0.0821
*A*_3_	[0.3750 0.1250 0.1250 0.3750]	4.0000	0.0000	0.89	0.0000
*A*_4_	[0.6250 0.2384 0.1365]	3.0183	0.0091	0.52	0.0176
*A*_5_	[0.5278 0.3325 0.1396]	3.0536	0.0268	0.52	0.0520

### Evaluation

The first level indicator set U = {u_1_, u_2_, …, u_n_,}, represents the criterion for the transformation capacity of the steel-based cities; while the second level set U_i_ = {u_i1_, u_i2_, … u_in_}, i = 1,2,… s contains the s factors associated with U and represents the indicator level of the transformation capacity of the city. A fuzzy relation matrix R_i_ for the set U_i_ can be built using the frequency of the evaluation values.

$$R_{i}=\left[\begin{array}{cccc} r_{11} & r_{12} & \cdots & r_{1m}\\ r_{21} & r_{22} & \cdots & r_{2m}\\ \vdots & \vdots & \ddots & \vdots \\ r_{i1} & r_{i2} & \cdots & r_{sm} \end{array}\right]$$

As indicated previously, the evaluation set *V* = {*v*_1_, *v*_2_, … *v*_5_} represents “excellent”, “good”, “average”, “worse” and “bad”, respectively. The weight distribution of the factor set U_i_ into V is A_i_ and R_i_ is the evaluation matrix for each individual indicator. The second-level evaluation vector is *B*_*i*_ = *A*_*i*_ *R*_*i*_ = (*b*_i1_, *b*_i2_, *… b*_*im*_), *i = 1*,*2*, *…s*.

If *U*_*i*_ is seen as factor *K*, *K* = {*u*_1_, *u*_2_, …, *u*_s_}. The factor set and its evaluation vector are shown below:

$$R=\left[\begin{array}{c} B_{1}\\ B_{2}\\ \vdots \\ B_{s} \end{array}\right]=\left[\begin{array}{cccc} b_{11} & b_{12} & \cdots & b_{1m}\\ b_{21} & b_{22} & \cdots & b_{2m}\\ \vdots & \vdots & \ddots & \vdots \\ b_{s1} & b_{s2} & \cdots & b_{sm} \end{array}\right]$$

As *U*_*i*_ is part of U, the first-level evaluation vector *B = A R* = (*b*_1_, *b*_2_, … *b*_*m*_) and is calculated through the index weights in the AHP.

### Evaluated results

According to the maximum achieved evaluation value, the transformation capacity of the selected resource-based cities is represented by *V* = *Max*(*b*_1_, *b*_2_, … *b*_*m*_). The T1, T2 and T4 indicators for the selected cities are assessed based on 2000–2013 data with the evaluation values determined using the corresponding fuzzy sets. For the T3 indicators, the evaluation values are decided by EEM.

The above methodology is applied for two Chinese steel-based cities, namely Panzhihua, Sichuan province (administratively a prefecture-level city) with an estimated population of 630,000 in 2010 [50], and Daye, Hubei province (administratively a county-level city of Huangshi City) with an estimated population of 450,000 in 2010 [51].

## Case Studies

The two case studies of Panzhihua and Daye were selected as examples of how to evaluate the capacity of steel-based cities for industrial restructuring, transformation and change of direction. Located in West China, Panzhihua’s construction started in 1965 and the city was the country’s first special zone for resource development; it is now a major producer of iron and steel, together with other resources such as vanadium, titanium and energy, including hydropower [52]. Located in east-central China, modern Daye was re-established in 1962 (a city with a similar name has existed on and off in Hubei province for centuries) around the production of steel and thermal power generation; it also has copper deposits, a large chemical fertilizer plant and textile mills for locally grown cotton [53]. Both cities are historically steel-based centres and now looking for industrial transformation. Panzhihua is a major centre for western and Daye for central China.

### Panzhihua: pillar industry transformation capacity

Using statistical data from China’s Statistical Yearbooks [54], China Industry Economy Statistical Yearbooks [42], China City Statistical Yearbooks [55] and Panzhihua Statistical Yearbooks [56], and the indicators from Table 1, a FRM [57,58] of the primary pillar industrial development capacity was constructed as follows:

$$R_{6\times 5}^{\prime }=\left[\begin{array}{ccccc} 0.0909 & 0.4545 & 0.4546 & 0.0000 & 0.0000\\ 0.0769 & 0.3846 & 0.2308 & 0.1538 & 0.1538\\ 0.8333 & 0.5000 & 0.1667 & 0.2500 & 0.0000\\ 0.8333 & 0.1667 & 0.2500 & 0.3333 & 0.1667\\ 0.2000 & 0.3000 & 0.4000 & 0.1000 & 0.0000\\ 0.2000 & 0.3333 & 0.6667 & 0.0000 & 0.0000 \end{array}\right]$$

The evaluation vector is

$$B^{\prime }=A_{1\times 6}^{\prime }R_{6\times 5}^{\prime }\;=\;\left[0.0804\;0.4008\;0.3040\;0.1654\;0.0494\right]$$

Similarly, the sub-factors set FRM of industrial transform capacity according to Table 2 can be constructed, then multiplied by the weight vector and then obtain the sub-factors U_i_ and evaluation vectors B_i_, which represent the fuzzy evaluation matrix (FEM) of U:

$$R_{5\times 5}^{\prime \prime }=\left[\begin{array}{ccccc} 0.1068 & 0.3646 & 0.3432 & 0.1682 & 0.0170\\ 0.0769 & 0.4136 & 0.3003 & 0.0869 & 0.0000\\ 0.0000 & 0.1944 & 0.5972 & 0.1181 & 0.0903\\ 0.2272 & 0.4488 & 0.2926 & 0.0199 & 0.114\\ 0.4689 & 0.3204 & 0.1374 & 0.0741 & 0.0000 \end{array}\right]$$

We then obtain the vector of the pillar industry’s transformation capacity:

$$R_{8\times 5}^{\prime \prime \prime }=\left[\begin{array}{ccccc} 0.1667 & 0.4167 & 0.3333 & 0.0833 & 0.0000\\ 0.1667 & 0.3333 & 0.4167 & 0.0000 & 0.0833\\ 0.0909 & 0.4545 & 0.2727 & 0.1818 & 0.0000\\ 0.1538 & 0.3077 & 0.3846 & 0.0769 & 0.0769\\ 0.0909 & 0.3636 & 0.3636 & 0.0909 & 0.0909\\ 0.1667 & 0.2500 & 0.3333 & 0.1667 & 0.0833\\ 0.1538 & 0.4615 & 0.2380 & 0.0769 & 0.0769\\ 0.0833 & 0.5000 & 0.3333 & 0.0833 & 0.0000 \end{array}\right]$$

Panzhihua’s evaluation vector is:

$$B^{\prime \prime }=A_{1\times 5}^{\prime \prime }R_{5\times 5}^{\prime \prime }\;=\;\left[0.1739\;0.3556\;0.3432\;0.1067\;0.0207\right]$$

According to Table 3, the FRM of for the substitute industries’ capacity is:

$$B^{\prime \prime \prime }=A_{1\times 8}^{\prime \prime \prime }R_{8\times 5}^{\prime \prime \prime }\;=\;\left[0.1315\;0.3562\;0.3427\;0.1108\;0.0587\right]$$

Using the maximum evaluation level, the assessment of Panzhihua’s pillar industry’s development capacity, namely that of the steel industry, is 0.4008 which corresponds to “good”; its industrial transformation capacity is 0.3556, also corresponding to “good” and substitute industries’ capacity is 0.3562, assessed similarly as “good”.

### Daye: pillar industry transformation capacity

Using statistical data from China’s Statistical Yearbooks [54], China Industry Economy Yearbooks [42], China City Statistical Yearbooks [55] and Daye Statistical Yearbooks [59], and the indicators from Table 1, the evaluation vectors for Daye are as follows:

$$B^{\prime }=A_{1\times 6}^{\prime }R_{6\times 5}^{\prime }\;=\;\left[0.0498\;0.1892\;0.3317\;0.3070\;0.1223\right]$$

$$R_{5\times 5}^{\prime \prime }=\left[\begin{array}{ccccc} 0.0748 & 0.3033 & 0.3624 & 0.2362 & 0.0196\\ 0.0642 & 0.1825 & 0.4707 & 0.2280 & 0.0545\\ 0.0000 & 0.0593 & 0.4797 & 0.3326 & 0.1284\\ 0.1023 & 0.1724 & 0.5842 & 0.1240 & 0.052\\ 0.2591 & 0.3928 & 0.2326 & 0.1081 & 0.0082 \end{array}\right]$$

B″ = [0.0819 0.2519 0.4261 0.2265 0.0498]

B′′′ = [0.0918 0.3106 0.4210 0.1142 0.624]

The assessment of the pillar industry’s development capacity is 0.3317 or “average”; its industrial transformation capacity is 0.4261 or “average” and its substitute industries’ capacity is 0.4210 or “average” again.

### Comparison between Panzhihua and Daye

As theory suggests resource-based cities reflect the life cycle of their industry, which is from construction to recruitment, transition, maturity, decline and closure. Hence, it is likely that some steel-based cities in China will experience decline with the life cycles of the industry itself unless they are capable of industrial transformation.

**Fig 2:**
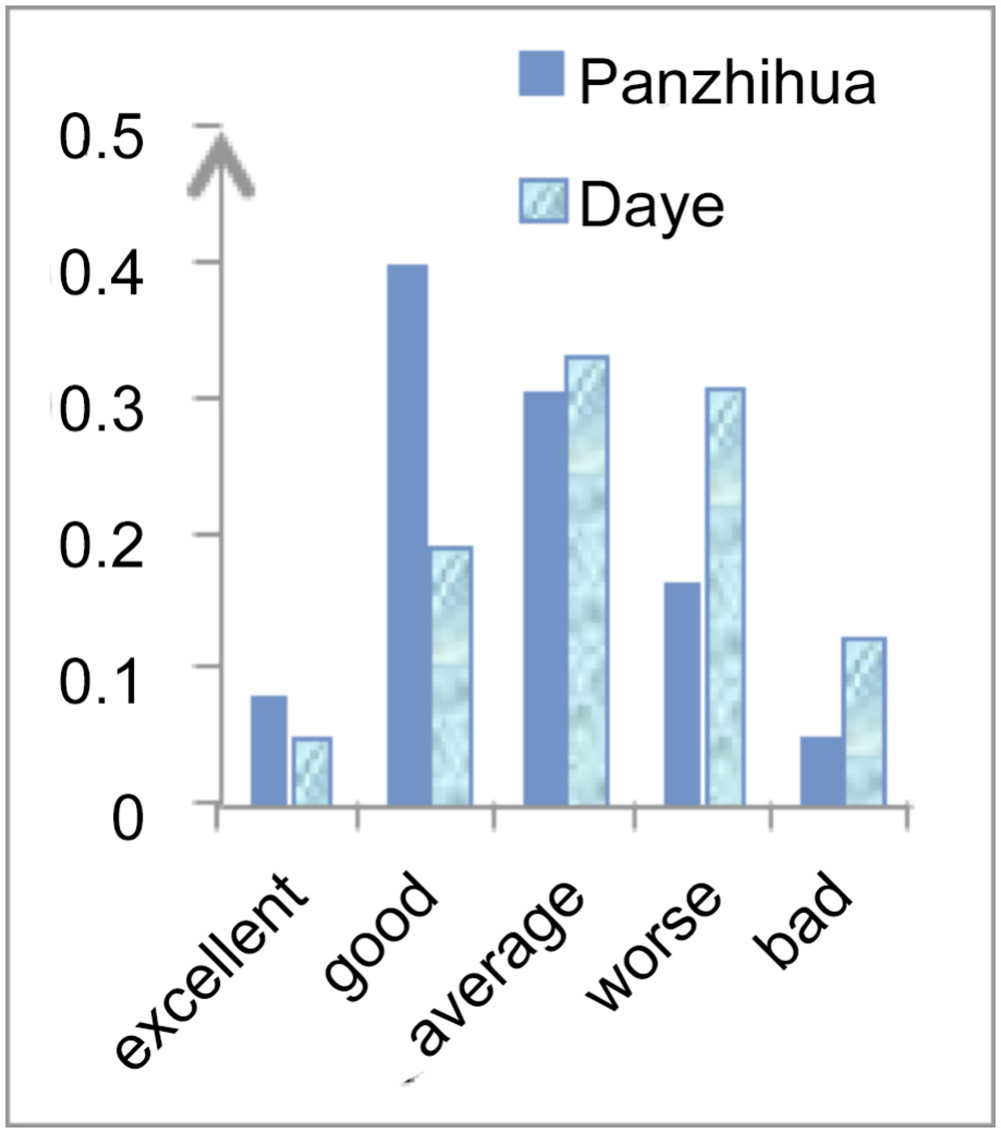
Existing Pillar Industry’s Improvement Capacity.

**Fig 3:**
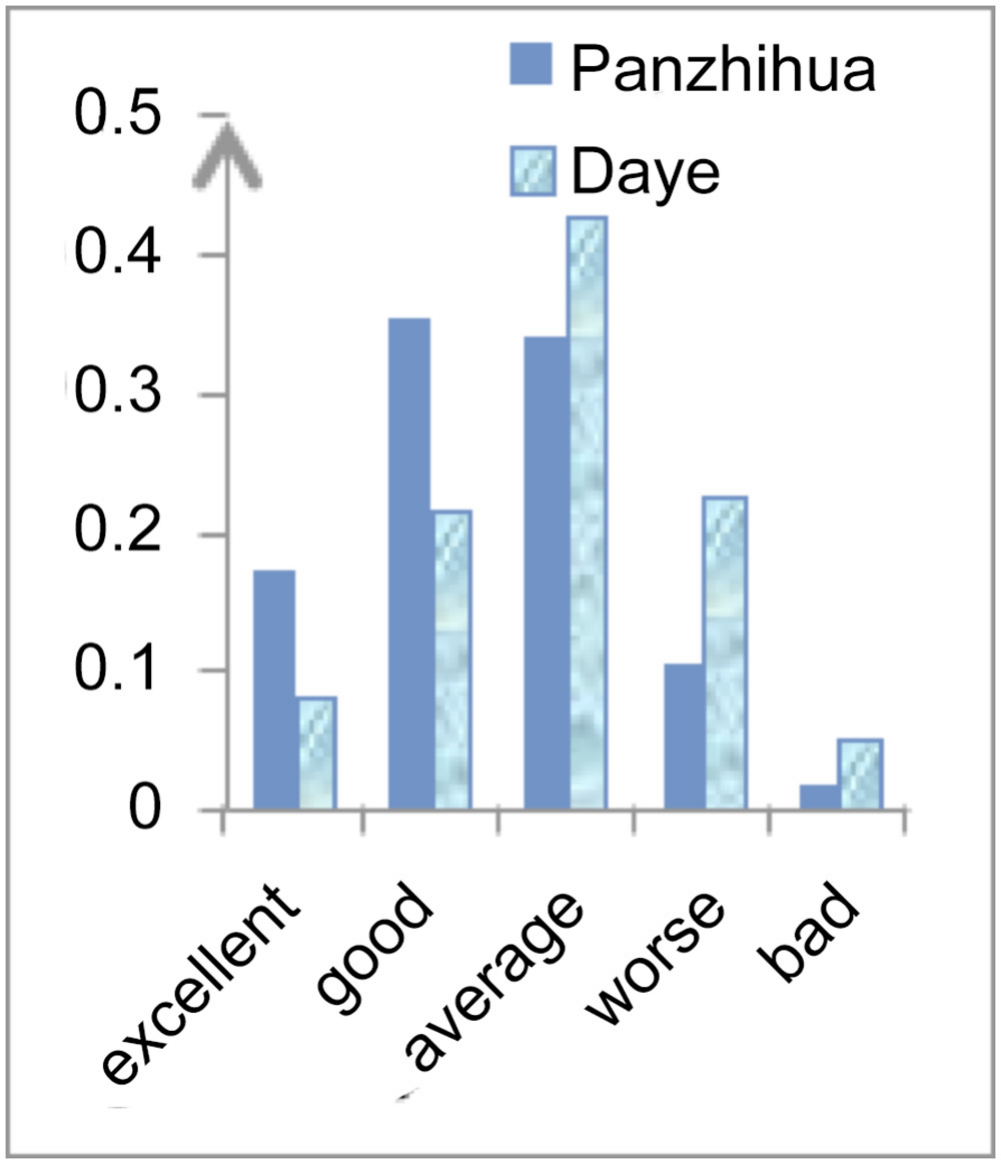
Existing Pillar Industry’s Transformation Capacity.

**Fig 4:**
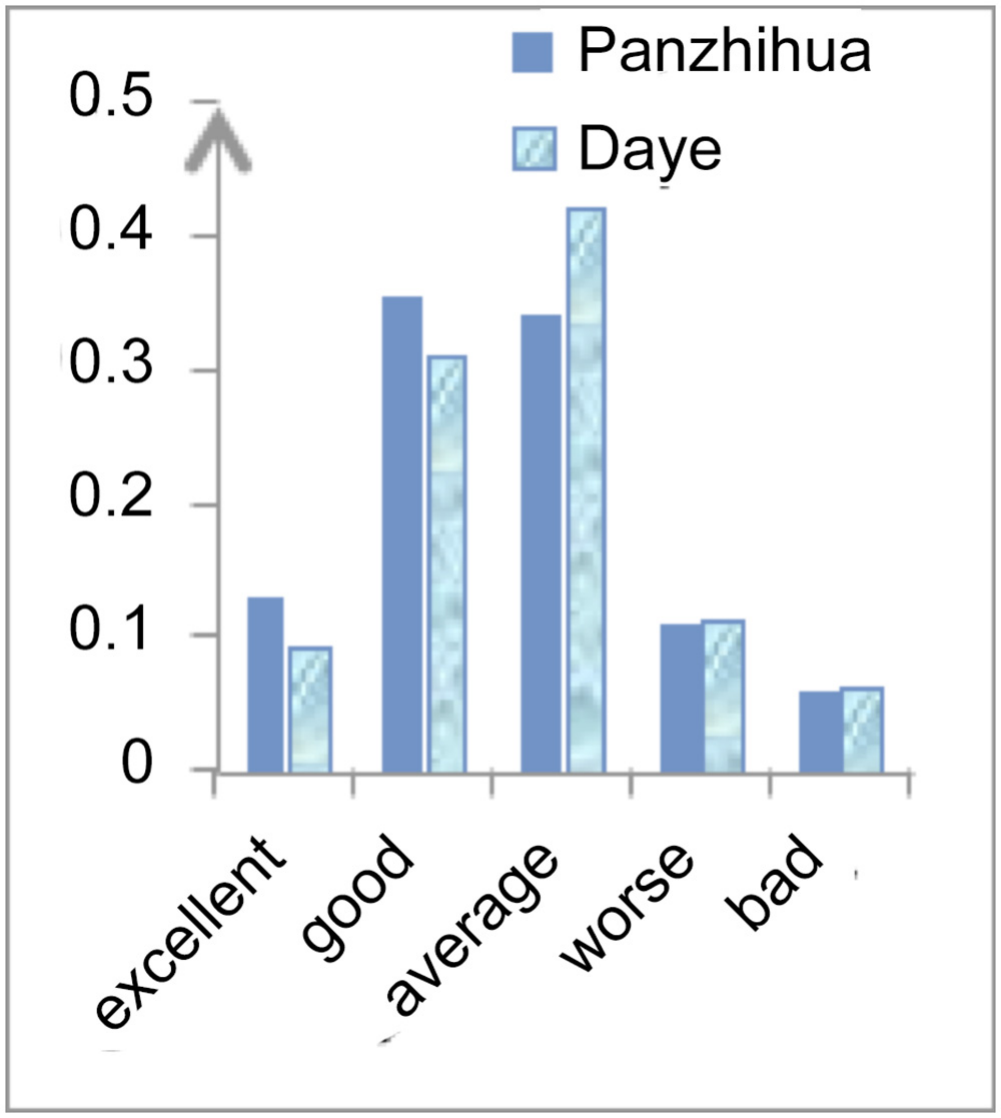
Substitute Industries’ Development Capacity.

Figs 2–4 show a comparison between Panzhihua and Daye and differences in their capacity for industrial transformation. The biggest differences between Panzhihua and Daye are in the pillar industry’s continuous improvement capacity and industry transformation capacity. Both are “good” for Panzhihua but “average” for Daye. Although the improvement capacity for the substitute industries is “good” for Panzhihua and “average” for Daye, the differences between the actual figures are small. The main indicators for these differences are mineral reserves, foreign capital investment, output of high-tech industries and rate of profit in the existing pillar industry. There is very little that a government can do in relation to mineral reserves and the profit of the existing pillar industry, however it can encourage foreign investment and high-tech industries development. The local governments of Panzhihua and Daye can support the development of high-tech industries and facilitate the flow of foreign capital by using the experience of mature cities, with the aim to improve the industry transformative capacity of these steel-based cities.

According to China’s *Sustainable Development Plan of Resource-based Cities 2013–2020* [2], Panzhihua is a mature while Daye is a declining steel-based city. The results of the evaluation from the current study confirm this classification and raise concerns about the cities’ actual situation, particularly in the case of Daye. Therefore, it is necessary for policy makers to encourage and establish targets for resilient infrastructure, enhance comprehensive and sustainable industrialization and foster innovation, particularly for resource declining cities like Daye. These are important aspects of the forthcoming Sustainable Development Goals framework [60].

## Conclusion and Policy Recommendations

Using a combination of Fuzzy Comprehensive Evaluation, including improved Fuzzy Relation Matrix, and Analytic Hierarchy Process techniques, this study developed a new evaluation method with a set of indicators and assessment values. This method was applied to evaluate the industrial transformation capacity of two steel-based Chinese cities—Panzhihua and Daye from a two-stage perspective, namely (1) their capacity to continue with the current pillar industry and (2) their capacity to transition to new substitute industries. Industrial transformation is required not only because of resource depletion but also due to pollution associated with steel and other heavy industries [61].

Panzhihua was found to be better suited not only for continuing with the existing steel industry but also to transform itself and diversity with substitute industries. Despite Daye facing a declining steel-industry situation, the city has not been able to generate conditions that would allow new substitute industries to flourish. There is however potential to put policies in place which encourage not only transformation but also sustainable production ways, such as industrial ecology, low carbon and circular economy [33,62] to achieve a resource efficient and environmentally friendly society [63].

Despite overall performing better, Panzhihua should make concerted efforts to transform its steel-based economy towards more sustainable and greener industries through increasing the proportion of new substitute industries. The existing but non-dominating substitute industries need to be properly placed within the municipal economy with government support but also through enterprise self-management and technological innovation.

Compared to Panzhihua, Daye faces more serious transformation barriers. Its local economy needs to develop new emerging industries in a low carbon circular economy rather than fully depend on its limited natural resources. In order to achieve sustainable transition, both local governments need to adjust their economic environments to encourage foreign investment and support high-tech and sustainable industry development. The experience from other European cities shows that public funding, leadership and infrastructure investment are crucial in encouraging private initiative and enterprises [13]. Public support and funding are also essential for transforming China’s resource-based cities into more sustainable and resource efficient economic centres.

## Supporting Information

S1 Table List of articles used for text mining. (XLSX)

S2 Table 200 keywords derived from the data mining process. (DOCX)

S3 Table Text mining expert’s review of keywords. (DOCX)

S4 Table Resource-based city expert’s review of keywords (DOCX)

S5 Table Investment expert’s review of keywords. (DOCX)

S6 Table Indicator values. (XLSX)

S7 Table Values for T1 indicators, 2000–2013. (DOCX)

S8 Table Indicator weights. (XLSX)

S9 Table Correlation between indicators. (XLSX)

S10 Table Fuzzy relation matrix, Panzhihua. (XLSX)

S11 Table Fuzzy evaluation matrix, Panzhihua. (XLSX)

S12 Table Pillar industry’s transformation capacity, Panzhihua. (XLSX)

S13 Table Fuzzy relation matrix, Daye. (XLSX)

S14 Table Fuzzy evaluation matrix, Daye. (XLSX)

S15 Table Pillar industry’s transformation capacity, Daye. (XLSX)

S16 Table Existing pillar industry’s improvement capacity, Panzhihua and Daye. (XLSX)

S17 Table Existing pillar industry’s transformation capacity, Panzhihua and Daye. (XLSX)

S18 Table Substitute industries’ development capacity, Panzhihua and Daye. (XLSX)
