# Supplementary material for: Evaluating Pillar Industry’s Transformation Capability: A Case Study of Two Chinese Steel-Based Cities
Source: PLoS One. 2015 Sep 30;10(9):e0139576. doi: 10.1371/journal.pone.0139576 (PMC4589354; doi:10.1371/journal.pone.0139576)
Supplement: S2 Table — (DOCX) [file pone.0139576.s002.docx]

**S2. Table. 200 keywords derived from the data mining process.**

1.改革开放: Reform and Opening-up

2.钢铁: Steel

3.接替: Substituted

4.城市: City

5.主导产业: Pillar

6.产业: Industry

7.高新技术: Hi-tech

8.水: Water

9.废气: Effluent and waste gas

10.废物: Solid Waste

11.资源: Resource

12.结构: Structure

13.产业链: Industrial chain

14.投资: Investment

15.人力: Human resources

16.新兴: Emerging

17.粗放型: Extensive

18.浪费: Waste

19.优势: Regional

20.产值: Output value

21.转型: Transformation

22.生命周期: Life cycle

23.创新: Innovation

24.政策: Policy

25.传统: Traditional

26.市场: Market

27. 要素: Production

28.优惠: Preferential

29.融资: Financing

30.基金: Funding

31.优化: Optimization

32.效率: Efficiency

33.企业: Enterprise

34.增长: Growth

35.污染: Pollution

36.治理: Abatement

37.人均: Per-capita

38.储蓄: Savings

39.教育的: Educational

40.科研: Research and Development (R&D)

41.内需: Domestic demand

42.资产: Assets

43.第二: Secondary

44.能耗: Energy consumption

45.绿色经济: Green economy

46.协同: Collaboration

47.就业: Employment

48.法规: Regulations

49.财政: Finance

50.税收: Tax

51.整合: Integration

52.发展: Development

53.利润率: Profit rate

54.创新力： Creativity

55.技术改造: Technology upgrade

56.阶段: Phase

57.指标: Indicator

58.衰退: Recession

59.变化: Change

60.风险: Venture

61.国企改革: State-owned enterprise

62.机械: Machinery

63.调研: Investigate and survey

64.多元化: Diversification

65. 中国: China

66.产品: Product

67.矿业: Mining

68.开采: Exploitation

69.稀缺性: Scarcity

70.配置: Allocation

71.恶性: Vicious

72.人口: Population

73.工具: Instrument

74.诅咒: Curse

75.优势: Advantage

76.分开: Separation

77.延伸: Extension

78.动力: Dynamics

79.沉淀: Sunk

80.生态: Ecology

81.社会: Society

82.公共: Common

83.问题: Problem

84.管辖: Jurisdiction

85.瓶颈: Bottleneck

86.方式: Mode

87.失业: Unemployment

88.压力: Pressure

89.园区: Park

90.资金: Capital

91.综合: Integrated

92.保障: Security

93.福利: Welfare

94.贷款: Loan

95.存款: Saving

96.新能源: New energy

97.经济: Economy

98.可持续发展: Sustainability

99.设施: Infrastructure

100.外商: Foreign

101.效率: Efficiency

102.科学: Scientific

103.贡献: Contribution

104.股权: Shareholder rights

105.债券: Bond

106.关联: Relationship

107.利用: Utilization

108.民营: Privately owned

109.资金: Capital

110.过度: Transition

111.空间: Space

112.耦合: Coupling

113.积累: Accumulation

114.产业: Tourist

115.障碍: Barrier

116.自然: Nature

117.挑战: Challenge

118.探索: Exploration

119.研究: Research

120.标准: Standard

121.执行: Execution

122.标杆: Model

123.系统: System

124.国家: Country

125.集中: Centralization

126.单位: Department

127.模式: Pattern

128.聚集: Aggregation

129.调整: Adjustment

130.背景: Background

131.计划: Plan

132提升: Raising

133.成果: Achievement

134.园区: Park

135.生态: Ecology

136.西部: Western

137.二氧化硫: Sulfur dioxide

138.烟尘: Dust and fume

139.电子: Electronics

140.技术: Technology

141.中部: Center

142.新兴: Emerging

143.海洋: Ocean

144.超效率: Ultra-efficiency

145.前沿: Frontier

146.中国: China

147.东北: Northwest

148. 减排: Emission reduction

149.信息: Information

150.崛起: Rising

151.第三: Tertiary

152. 加拿大: Canada

153.天然气: Natural gas

154.汽车: Automobile

155总量: Total volume

156我国: Domestic

157.非金属: Non-metal

158.纤维: Fiber

159.土壤: Soil

160.城镇: Town

161.职能: Function

162核心: Core

163边缘: Edge

164鲁尔: Ruhr

165匹兹堡: Pittsburgh

166大阪: Osaka

167原材料: Raw materials

168商品: Stock

169建设: Construction

170欧洲: Europe

171地理: Geographic

172收入: Income

173成熟: Maturity

174归属感: Sense of belonging

175困难: Difficulty

176第三: Third

177依附: Dependency

178理论: Theory

179密集: Intensive

180柔性: Flexibility

181劳动力: Labor

182基地: Base

183分类: Classification

184制度: System

185规模: Scale

186单一: Single

187安全: Safety

188:教育： Education

189扶持: Support

190垄断: Monopoly

191平衡: Balance

192关系 Relationship

193跨越: Leapfrog

194规律: Pattern

195时间: Time

196预警: Forewarning

197防治: Prevention

198自由: Freedom

199建设: Construction

200互补： Complementation
