# Supplementary material for: Evaluating Pillar Industry’s Transformation Capability: A Case Study of Two Chinese Steel-Based Cities
Source: PLoS One. 2015 Sep 30;10(9):e0139576. doi: 10.1371/journal.pone.0139576 (PMC4589354; doi:10.1371/journal.pone.0139576)
Supplement: S3 Table — (DOCX) [file pone.0139576.s003.docx]

**S3. Table. Text mining expert’s review of keywords**

改革开放 reform and opening-up

主导产业 pillar industry

水污染 water pollution

废气排放 waste gas emissions

固体废物 solid waste

自然资源 natural resource

产业结构 industrial structure

产业链 industrial chain

投资 investment

生命周期 life cycle

增长速度 growth rate

融资渠道 financing source

政策支持 policy support

教育程度 education level

科研经费 R&D funding

内需拉动 internal demand motivation

固定资产 fixed assets

工业污染 industrial pollution

污染治理 pollution control

绿色经济 green economy

发展前景 development prospect

技术改造 technology upgrade

经济体制 economic system

生态 ecology

社会职能 social function

公共资源 common resource

社会问题 social issues

失业 unemployment

综合利用 integrated usage

社会保障 social security

福利 welfare

可持续发展 sustainability

基础设施 basic infrastructure

信息产业 information industry

科学研究 scientific research

贡献率 contribution rate

信息产业 information industry

股权 shareholder’s right

债券 bond

产业关联 industrial connection

城市转型 city transformation

资金浪费 funding waste

空间分布 space distribution

耦合coupling

发展障碍 development barriers

自然条件 natural condition

探索 exploration

研究 research

标准 standard

二氧化硫 S0_2_

烟尘 soot

信息技术 information technology

新兴产业 new industry

减排 emission reduction

信息产业 information industry

崛起 rising

天然气 natural gas

总量 total

土壤 soil

职能 function

核心 core

原材料 raw material

建设 construction

第三次科技革命 third technology revolution

技术密集 technology-intensive

劳动力 labor force

分类 classification

制度 system

安全 safety

科教science education

扶持 aid

平衡 balance

规律 rule

预警 warming

防治 prevention
