# Supplementary material for: Evaluating Pillar Industry’s Transformation Capability: A Case Study of Two Chinese Steel-Based Cities
Source: PLoS One. 2015 Sep 30;10(9):e0139576. doi: 10.1371/journal.pone.0139576 (PMC4589354; doi:10.1371/journal.pone.0139576)
Supplement: S4 Table — (DOCX) [file pone.0139576.s004.docx]

**S4. Table. Resource-based city expert’s review of keywords**

钢铁资源（储量，利用效率等）资源产品、资源开采 steel resource (reservation, using efficiency) resource product, resource extraction

钢铁产业（先进程度，产值等）steel industry (level of advancement, output value)

接替产业（技术、产值、适应性等）substitute Industry (technology, output value, suitability)

主导产业（占比、产值、技术、地位等）pillar industry (percentage, output value, technology, position)

高新技术（先进性）high-tech (level advancement)

水污染water pollution

废气排放 waste gas emissions

固体废物 solid waste discharge

自然资源（资源种类、储量、可开发性）natural resource (types of resources , reservevation, exploitable)

产业结构（是否均衡）industrial structures (if balanced)

产业链（上下游衔接）industrial chain (connections)

投资（资金投向及效率）investment (investment target and efficiency)

人力资源（人才、各类技术工人等）human resource (talents, technicians)

新兴产业 new industries

资源浪费 resource waste

区位优势 location advantage

产值 output value

转型 transformation

生命周期（产业生命周期、产品生命周期）life cycle (industry life cycle, product life cycle)

培育创新（投入情况）developing innovation (implementation situation)

政策支持（财政、税收、金融货币、政府购买） policy support (finance, tax, financing monetary, government purchasing)

传统产业 traditional industry

市场机制 market mechanism

生产要素 production factor

优惠政策、法律法规、财政补贴、税收优惠、创新基金preferential policies , laws and regulations, subsidies , tax incentives, innovation fund

融资渠道 financing source

生产效率 productivity

企业数（各类企业数量）number of enterprises (number of all types of enterprises)

增长速度（经济、人口等社会发展指标）growth rate (economic, population and other social development indicators)

工业污染、能耗 industrial pollution, energy consumption

污染治理（治理投入、治理效果）pollution control (control input, control outcome)

人均产值 per capita output value

储蓄 saving

教育程度、科研经费、内需拉动 education level, R&D funding, domestic demand

固定资产 fixed assets

绿色经济（产值、发展条件、发展规划等）green economy (output value, development condition, developmental strategies)

协同、整合（城市系统产业的协调能力）collaboration, integration (integration ability of city’s industries)

就业人数 employment number

发展前景 development prospect

利润率 profitability

创新力、技术改造 innovation ability, technology upgrade

发展阶段 development stage

衰退（衰退指数）decline (declining index)

国企改革、多元化、经济体制、政企分开 state enterprise reform, diversification, economic system, government-enterprise separation

人口增长 population growth

金融工具 financial instrument

资源诅咒 resource curse

产业延伸 industry extension

内部动力 internal motivation

沉淀成本 sunk cost

生态 ecology

社会职能 social function

公共资源 common resource

发展瓶颈 development bottleneck

管理方式 management pattern

就业压力、失业 employment pressure, unemployment

综合利用（资源、人才等生产要素）integrated utilization (resource, talents and other production factors)

社会保障 social security

经济效益 economic efficiency

基础设施 basic infrastructure

外商投资 foreign investment

科学研究 science and technology

贡献率 contribution rate

产业关联（产业转型的关联产业） industrial linkage (linked industries to industrial transformation)

民营经济 private economy

耦合（关键指标的耦合关系）coupling (coupling of key indicators)

资本积累 capital accumulation

发展障碍 development barrier

自然条件 natural condition

系统（产业整合能力与产业演化动力）system (industrial integrated capacity and industrial evolutionary motivation)

模式（发展模式评价）mode (development mode evolution)

聚集（产业聚集度）clustering (industrial clustering level)

背景 background

融资 financing

投资 investment

园区（产业园区建设）zone (industrial zone development)

生态（生态经济与生态保护程度）ecology (ecological economy and ecological preservation)

二氧化硫、烟尘、减排 SO2, soot, emission reduction

信息技术、新兴产业、信息产业 information technology, new industry, information industry

超效率（各产业效率衡量）supremum efficiency (industrial efficiency measurement)

第三产业 the third industry

天然气（新能源）natural gas (new energy)

汽车 automobile

总量（总量判断）total value (volume level)

原材料raw material

地理特征 geographical characteristics

人均收入 per capita income

成熟（产业成熟度）mature (industrial mature level)

技术密集（技术要求程度） technology intensity (technology demand)

劳动力（人力资源之一） labour force

分类（产业分类、城市分类、指标分类）classification (industrial classification

安全（转型的安全性）safe (transformation safety)

扶持 aid

垄断（资源的垄断性）monopoly (monopolization of resources)

平衡（平衡发展度）balance (level of balanced development)

政企关系（经济体制）relationship between government and enterprises (economic system)

自由（资源市场配置自由度）freedom (freedom level of resource market configuration)

互补（产业间的互补性）complementary ( complementarity between industries )
