# Supplementary material for: Evaluating Pillar Industry’s Transformation Capability: A Case Study of Two Chinese Steel-Based Cities
Source: PLoS One. 2015 Sep 30;10(9):e0139576. doi: 10.1371/journal.pone.0139576 (PMC4589354; doi:10.1371/journal.pone.0139576)
Supplement: S5 Table — (DOCX) [file pone.0139576.s005.docx]

**S5. Table. Investment expert’s review of keywords**

钢铁资源 steel resource

主导产业 pillar industry

钢铁产业 steel industry

高新技术 high-tech

自然资源 natural resource

投资 investment

人力资源 human resource

新兴产业 new industry

资源浪费 resource waste

培育创新 cultivated innovation

政策支持 policy support

市场机制 market mechanism

生产要素 productivity factor

优惠政策preferential policy

融资渠道 financing channel

创新基金 innovation funding

优化配置optimized allocation

生产效率 productivity

企业数 number of enterprises

增长速度 growth rate

人均产值 GDP per capita

能耗 energy consumption

法律法规 law and regulation

财政补贴 financial compensation

税收优惠preferential taxation

发展前景 development prospect

利润率 profit rate

创新力 innovation capacity

技术改造 technology upgrade

风险资本 risk capital

国企改革 state enterprise reform

调研 investigation

多元化 diversification

经济体制 economic system

资源开采 resource exploitation

稀缺性 scarcity

恶性循环 vicious circle

金融工具 financing tool

优势 advantage

政企分开 separation between government and enterprise

产业延伸 industrial extension

生态 ecology

发展瓶颈 development bottle neck

管理方式 management pattern

资金 capital

综合利用 integrated usage

银行贷款 bank loan

新能源 new energy

经济效益 economic profit

可持续发展 sustainable development

外商投资 foreign investment

贡献率 contribution rate

股权 shareholder’s right

债券 bond

产业关联 industrial relation

民营经济 private economy

资金浪费 capital waste

资本积累 capital accumulation

自然条件 natural condition

问题 problem

探索 exploration

研究 research

标准 standard

调整 adjustment

融资 financing

投资 investment

二氧化硫 SO2

信息技术 information technology

新兴产业 new industry

减排 emission reduction

人均收入 per capita income

第三次科技革命 the third technology revolution

劳动力 labour force

制度 system

规模 size

科教 science and education

扶持 aid

垄断 monopoly

政企关系 relationship between government and enterprise

跨越 jump
