# Supplementary material for: Evaluating Pillar Industry’s Transformation Capability: A Case Study of Two Chinese Steel-Based Cities
Source: PLoS One. 2015 Sep 30;10(9):e0139576. doi: 10.1371/journal.pone.0139576 (PMC4589354; doi:10.1371/journal.pone.0139576)
Supplement: S7 Table — (DOCX) [file pone.0139576.s007.docx]

**S7. Table. Values for T1 indicators, 2000–2013**

| T1 | Index | 2013 | 2012 | 2011 | 2010 | 2009 | 2008 | 2007 | 2006 | 2005 | 2004 | 2003 | 2002 | 2001 | 2000 |
| --- | --- | --- | --- | --- | --- | --- | --- | --- | --- | --- | --- | --- | --- | --- | --- |
| $a_{11}$（%） | $Q_{0}$ | 7.4 | 8.1 | 8.7 | 10.2 | 8.3 | 10.1 | 14.6 | 13.3 | 10.1 | 10 | 9.1 | 8.3 | 8.4 | 7.6 |
|  | $\mathrm{Max}\left( Q_{i} \right)$ | 8.91 | 11.60 | 11.61 | 14.37 | 14.21 | 17.96 | 19.09 | 20.86 | 18.31 | 15.43 | 16.30 | 13.95 | 13.79 | 12.88 |
|  | $\mathrm{Min}\left( Q_{i} \right)$ | 3.15 | 4.25 | 5.77 | 8.08 | 0.57 | 7.02 | 9.16 | 9.93 | 7.79 | 6.30 | 7.05 | 6.21 | 6.05 | 3.94 |
| $a_{12}$（Yuan） | $Q_{0}$ | 41908 | 38459 | 35198 | 30015 | 25608 | 23708 | 20169 | 16500 | 14185 | 14185 | 12336 | 10542 | 9398 | 8622 |
|  | $\mathrm{Max}\left( Q_{i} \right)$ | 99607 | 93173 | 85213 | 76074 | 69164 | 66932 | 62041 | 54858 | 49649 | 44839 | 38486 | 33958 | 31799 | 30047 |
|  | $\mathrm{Min}\left( Q_{i} \right)$ | 22922 | 19710 | 16413 | 13119 | 10971 | 9855 | 7878 | 6305 | 5394 | 4317 | 3701 | 3257 | 3000 | 2759 |
| $a_{14}$（Yuan） | $Q_{0}$ | 32894 | 29508 | 25505 | 22619 | 19541 | 16407 | 13058 | 12293 | 10787 | 9197 | 8018 | 6766 | 5780 | 5076 |
|  | $\mathrm{Max}\left( Q_{i} \right)$ |  | 103454 | 94731 | 86662 | 78882 | 67492 | 54626 | 54376 | 48620 | 41007 | 36356 | 30848 |  |  |
|  | $\mathrm{Min}\left( Q_{i} \right)$ |  | 2852 | 2692 | 2610 | 2477 | 2431 | 2390 | 2261 | 2219 | 2243 | 2205 | 2193 |  |  |
| $a_{15}$（Yuan） | $Q_{0}$ |  |  | 1771.57 | 1458.85 | 1236.62 | 1091.91 | 919.41 | 746.71 | 643.86 | 557.17 | 480.42 | 426.62 | 363.38 | 303.69 |
|  | $\mathrm{Max}\left( Q_{i} \right)$ |  |  | 3652.23 | 3126.63 | 2843.78 | 2648.32 | 2432.75 | 2107.64 | 3398.69 | 3009.13 | 2698.33 | 2486.78 | 2304.85 | 1415.46 |
|  | $\mathrm{Min}\left( Q_{i} \right)$ |  |  | 1259.21 | 968.76 | 804.63 | 695.89 | 569.97 | 419.98 | 373.96 | 292.56 | 246.52 | 226.12 | 177.09 | 115.43 |
| $a_{16}$（Yuan） | $Q_{0}$ | 18023 | 16674 | 15161 | 13472 | 12265 | 11243 | 9998 | 8697 | 7943 | 7182 | 6511 | 6030 |  |  |
|  | $\mathrm{Max}\left( Q_{i} \right)$ | 28155 | 26254 | 25102 | 23200 | 20992 | 19398 | 17255 | 14762 | 13773 | 12631 | 11040 | 10464 |  |  |
|  | $\mathrm{Min}\left( Q_{i} \right)$ | 18023 | 16674 | 1259 | 969 | 805 | 696 | 570 | 420 | 374 | 293 | 247 | 226 |  |  |
| $a_{21}$ (number) | $Q_{0}$ |  |  | 0.1887 | 0.1869 | 0.1776 | 0.1806 |  |  |  |  |  |  |  |  |
|  | $\mathrm{Max}\left( Q_{i} \right)$ |  |  | 0.2799 | 0.2413 | 0.2678 | 0.1983 |  |  |  |  |  |  |  |  |
|  | $\mathrm{Min}\left( Q_{i} \right)$ |  |  | 0.0524 | 0.0517 | 0.4189 | 0.4317 |  |  |  |  |  |  |  |  |
| $a22$（%） | $Q_{0}$ | 0.0210 | 0.0199 | 0.0185 | 0.0177 | 0.0170 | 0.0146 | 0.0139 | 0.0139 | 0.0133 | 0.0123 | 0.0114 | 0.0108 | 0.0096 | 0.0091 |
|  | $\mathrm{Max}\left( Q_{i} \right)$ |  |  | 0.0576 | 0.0582 | 0.0551 | 0.05257 | 0.0543 | 0.0534 | 0.0551 | 0.0532 | 0.0514 |  |  |  |
|  | $\mathrm{Min}\left( Q_{i} \right)$ |  |  | 0.0041 |  |  | 0.0023 |  |  | 0.0021 | 0.0026 | 0.0017 | 0.002 | 0.0014 | 0.0015 |
| $a_{23}$（number） | $Q_{0}$ |  | 0.363 | 0.318 | 0.275 | 0.229 | 0.206 | 0.187 | 0.159 | 0.140 | 0.122 | 0.111 | 0.090 | 0.077 | 0.079 |
|  | $\mathrm{Max}\left( Q_{i} \right)$ |  | 0.759 | 0.633 | 0.495 |  |  |  |  |  |  |  |  |  |  |
|  | $\mathrm{Min}\left( Q_{i} \right)$ |  | 0.479 | 0.503 | 0.556 |  |  |  |  |  |  |  |  |  |  |
| $a_{24}$（%） | $Q_{0}$ |  | 1.0908 | 1.4631 | 1.6459 | 1.8478 | 2.9040 | 3.4036 | 3.6436 | 4.2063 | 4.4065 | 4.4345 | 4.6285 | 4.5561 |  |
|  | $\mathrm{Max}\left( Q_{i} \right)$ |  | 3.2347 | 4.0374 | 4.2388 | 4.9333 | 8.3894 | 9.0529 | 9.4716 | 10.8895 | 12.3491 | 11.2459 |  |  |  |
|  | $\mathrm{Min}\left( Q_{i} \right)$ |  | 0.9373 | 0.7865 | 0.2703 | 0.0000 | 0.0000 | 0.0606 | 0.0000 | 0.1694 | 0.0000 | 0.0329 |  |  |  |
| $a_{31}$wastewater（10 t/100mln Yuan） | $Q_{0}$ |  | 13.1819 | 13.9333 | 15.3733 | 17.2802 | 18.2037 | 20.9491 | 23.7839 | 28.3614 | 30.1735 |  |  |  |  |
|  | $\mathrm{Max}\left( Q_{i} \right)$ |  | 14.6939 | 14.7638 | 17.7714 | 22.6802 | 24.0762 | 28.0334 | 32.8075 | 38.3935 | 40.3277 |  |  |  |  |
|  | $\mathrm{Min}\left( Q_{i} \right)$ |  | 6.6804 | 7.6500 | 7.5375 | 7.8286 | 8.6615 | 9.7694 | 9.2344 | 18.3079 | 20.4275 |  |  |  |  |
| $a_{31}$waste emissions（t/100mln Yuan） | $Q_{0}$ | 35.9324 | 40.7652 | 46.8799 | 54.4229 | 64.9570 | 73.9129 | 92.8515 | 119.6776 | 137.8521 | 141.0385 |  |  |  |  |
|  | $\mathrm{Max}\left( Q_{i} \right)$ |  | 151.9347 | 193.6715 | 249.6284 | 300.4320 | 347.0389 | 476.7755 | 626.3414 | 677.1649 | 783.7645 |  |  |  |  |
|  | $\mathrm{Min}\left( Q_{i} \right)$ |  | 5.9693 | 6.8926 | 7.6006 | 3.7611 | 5.0652 | 5.5379 | 6.8785 | 8.0386 | 4.5384 |  |  |  |  |
| $a_{32}$ | $Q_{0}$ |  | 0.1786 | 0.1544 | 0.1417 | 0.1579 | 0.1876 | 0.1826 | 0.1559 | 0.1491 | 0.1126 |  |  |  |  |
|  | $\mathrm{Max}\left( Q_{i} \right)$ |  | 0.6777 | 0.4031 | 0.3647. | 0.3573 | 0.3649 | 0.2640 | 0.3628 | 0.3741 | 0.1657 |  |  |  |  |
|  | $\mathrm{Min}\left( Q_{i} \right)$ |  | 0.0580 | 0.1500 | 0.0813 | 0.0000 | 0.0000 | 0.0427 | 0.0275 | 0.0926 | 0.0733 |  |  |  |  |
| $a_{33}$（10t ton standard coal /100m Yuan） | $Q_{0}$ | 0.66 | 0.70 | 0.74 | 0.81 | 0.90 | 0.92 | 1.05 | 1.20 | 1.29 | 1.34 | 1.36 | 1.34 | 1.39 | 1.49 |
|  | $\mathrm{Max}\left( Q_{i} \right)$ |  |  | 2.28 | 3.31 | 3.45 | 3.69 | 3.95 | 4.1 | 4.14 |  |  |  |  |  |
|  | $\mathrm{Min}\left( Q_{i} \right)$ |  |  | 0.46 | 0.58 | 0.61 | 0.66 | 0.71 | 0.76 | 0.8 |  |  |  |  |  |

Source of data: compiled from *China Statistical Yearbook,* *Yearbook of Chinese Industry,* *China City Statistical Yearbook. Daye Statistical Yearbook* from 2000-201
